# Improving Pediatric Surgical Follow-Up Documentation: A Prospective Clinical Audit in the Department of Pediatric Surgery at National Ribat University Hospital, Sudan

**DOI:** 10.7759/cureus.81888

**Published:** 2025-04-08

**Authors:** Mohamed Y Ibrahim, Alshaima Koko, Arwa Abdelraouf Ahmed, Esra S Abdalgadir, Mohammed Alameen Genaid Malik Moheyaldeen, Salah Aldeen Alsheikh, Mohamed Khalafalla, Marwa Suliman, Mohamed Fadlalla, Mohamed Alghazali, Hussamaldin Hassan, Noura Ali, Almuaz Ali, Nafisa Ahmed, Ahmed H Elhussein

**Affiliations:** 1 Paediatric Surgery Center, National Ribat University Hospital, Khartoum, SDN; 2 Trauma and Orthopaedic Surgery, Ibrahim Malik Teaching Hospital, Khartoum, SDN; 3 Internal Medicine, National Ribat University Hospital, Khartoum, SDN

**Keywords:** clinical audit system, improving healthcare quality, pediatrics, pediatric surgery, quality improvement projects, surgery

## Abstract

Background: Proper clinical documentation is essential for patient safety and the continuity of care, especially in pediatric surgery. In a resource-limited setting at Ribat University Teaching Hospital in Khartoum, Sudan (2021-2022), this study looked at how well the B-SOAP (short for Background, Subjective, Objective, Assessment, and Plan) follow-up documentation structure was followed. Baseline audits identified significant flaws, with a 21.9% (21 out of 96) adherence rate, highlighting systemic shortcomings in organized documentation standards.

Materials and methods: We performed a prospective observational audit over three cycles (pre-intervention, intervention, and post-intervention), examining 68 B-SOAP sheets. Cause-and-effect diagrams and Pareto charts used in pre-intervention audits showed that the main problems were a lack of standard templates, poor training, and monitoring that wasn't consistent. Interventions comprised the revision of the B-SOAP template, the implementation of training sessions, and the establishment of audit feedback mechanisms. Compliance was evaluated by descriptive statistics, utilizing a 90% standard for completeness and correctness.

Results: Post-intervention compliance increased markedly to 90% (22 out of 24) (Δ+68.1%), exceeding objectives. The most significant improvement was seen in the Plan part, which went from 32.5% (seven out of 21) to 65% (14 out of 22). This was followed by the Subjective (21.2%, from four out of 21 to nine out of 22) and Assessment (21.0%, from two out of 21 to seven out of 22) parts. The documenting of objectives continued to be difficult (+16.4%), indicating ongoing obstacles to uniform data entry. Iterative audits and systematic feedback facilitated gradual improvements, consistent with evidence about the effectiveness of audits in resource-constrained environments.

Conclusion: Structured interventions, such as standardizing templates, training, and regular audits, greatly increased B-SOAP compliance, showing that it is possible to do so with paper-based systems. Despite problems with the infrastructure, the fact that 90% of the people who were supposed to follow the rules did so after the intervention shows how important it is to improve quality in a planned way. Maintaining progress necessitates continuous education, regular audits, and scalable digital solutions. This study gives a framework that can be used again and again to improve clinical documentation in similar settings with limited resources. This will immediately improve patient safety and care quality.

## Introduction

Record keeping is an essential element of healthcare. It maintains accuracy and accountability in patient management [1]. The B-SOAP format (short for Background, Subjective, Objective, Assessment, and Plan) provides a structured framework that aids in proper clinical documentation and organized communication as well as enhancing the quality of care [2]. In pediatric surgery, thorough follow-up documentation is vital. As referred to the complicated nature of cases and the susceptibility of this patient population. The effective use of the B-SOAP format ensures accurate record-keeping to enhance the integration of care and patient safety. Studies highlight that improving documentation practices comes hand in hand with better clinical outcomes, reduced medical errors, and enhanced interprofessional collaboration. Also, studies indicate that standardized documentation can reduce medical errors by up to 30% and improve interprofessional collaboration [1].

Evidence indicates that adherence to standardized documentation protocols exhibits variability across diverse healthcare environments, notwithstanding their critical importance. For example, significant discrepancies in the implementation of the SOAP format during pediatric ward rounds were noted, with educational initiatives and systematic audits identified as pivotal determinants for improving compliance [3]. Likewise, researchers clarified the difficulties encountered in the transition to electronic health records within pediatric care, accentuating the necessity for training to enhance clinical documentation practices [4]. Although frameworks such as B-SOAP are acknowledged on a global scale, their integration within resource-constrained environments frequently encounters distinct obstacles, including inadequate access to training and infrastructural limitations. In the context of Sudan, Elhadi Bakheet et al. [5] reported challenges pertaining to surgical documentation, emphasizing the need for audits and feedback mechanisms to rectify deficiencies in compliance. In resource-limited settings like Sudan, infrastructural constraints further exacerbate these issues, with surgical documentation audits showing omission rates as high as 60% for critical patient data [5,6].

Disregarding the significance of proper documentation poses substantial risks. Poor compliance with standardized formats such as B-SOAP may result in communication difficulties and delayed responses, leading to compromised patient safety [7]. In addition, it interferes with the capacity to perform effective audits and quality improvement programs, which are critical for enhancing care standards [8]. In pediatric surgery, improper documentation limits the ability to monitor patient outcomes, recognize systematic problems, and conduct patient-centered interventions. As Muhammed et al. noted, effective documentation practices are essential for assessing and enhancing the quality of inpatient care, especially in surgical specialties [9].

This study aims to audit the compliance of doctors with the B-SOAP follow-up sheet in the pediatric surgery department at Ribat University Teaching Hospital, Khartoum, Sudan, during 2021-2022. The hypothesis proposes that adherence to the B-SOAP format is insufficient, influenced by adjustable barriers including inadequate training or monitoring systems. The main aim is to assess compliance rates, investigate contributing factors, and suggest evidence-based recommendations for enhancing documentation practices. This paper aims to improve patient safety, facilitate effective communication, and promote quality care in pediatric surgery by addressing existing gaps and fostering a culture of accountability.

## Materials and methods

Study design

This study employed an audit methodology to assess compliance with the B-SOAP follow-up sheet among doctors in the pediatric surgery department at Ribat University Teaching Hospital between 2021 and 2022. A cross-sectional approach was used as it provides a clear idea of documentation practices at a given point [10]. Additionally, this design facilitated the identification of key areas for improvement and modification of the B-SOAP sheet to better align with the needs of a pediatric surgery department [11].

Data collection

The study was conducted in an inpatient setting. Data were collected using a Google Form (Google LLC, Mountain View, CA) designed to assess compliance with standardized follow-up guidelines and structured formats. The inclusion criteria comprised all pediatric surgery inpatients with long-stay files (stay more than 24 hours). Cases were excluded if documentation was incomplete due to patient discharge within 24 hours or if records were lost. The audit tool evaluated key elements of the B-SOAP framework, including completeness, accuracy, and relevance, with a standard of 90% for each category [3].

Each B-SOAP component was evaluated for completeness and accuracy using the following binary scale (1 = fully documented, 0 = missing/incomplete): Background: patient history, diagnosis, and relevant context; Subjective: patient-reported symptoms or caregiver inputs; Objective: measurable clinical findings (e.g., vitals, exam results); Assessment: clinical judgment/differential diagnosis; Plan: actionable management steps (e.g., medications, follow-up).

Pre-intervention phase

The audit began with a total of 21 forms of follow-up notes that were randomly chosen from the department, ensuring a simple randomization method. This analysis revealed numerous issues with the documentation and a 21.9% (21 out of 96) adherence to guidelines, falling significantly behind.

The pre-intervention B-SOAP sheet lacked a standardized template, leading to inconsistent documentation. The Background section often missed key patient history details, the Subjective section lacked patient-reported symptoms, the Objective section omitted critical clinical findings, the Assessment section was frequently incomplete, and the Plan section often lacked actionable management strategies.

Root cause assessment

The auditing team, under the supervision of both clinical and quality improvement offices, conducted root cause analysis by applying quality improvement tools such as the Ishikawa diagram, Pareto chart, and Plan, Study, Do, Act (PDSA) approach to tackle the main defect. The primary concerns identified in the current documentation practices included the absence of a standardized B-SOAP template and a lack of awareness among healthcare providers regarding the significance of comprehensive follow-up notes. Furthermore, the scarcity of clear guidelines resulted in frequent omissions of crucial details. These inconsistencies in documentation practices posed challenges to maintaining continuity of care, heightened the risk of miscommunication, and raised potential medico-legal issues. Addressing these gaps was essential to ensure that the B-SOAP follow-up sheet could facilitate accurate and structured documentation, particularly in pediatric surgical contexts.

Intervention phase 

We adopted the B-SOAP sheet as a foundational tool for documentation. Subsequently, we modified it to align with the specific needs of our pediatric surgical center, ensuring it captured essential patient progress details in a structured manner. The post-intervention B-SOAP sheet was revised to include a standardized template (Appendix A). The Background section was expanded to capture comprehensive patient history, the Subjective section was structured to include detailed patient-reported symptoms, the Objective section was enhanced to include all relevant clinical findings, the Assessment section was improved to ensure thorough clinical evaluation, and the Plan section was detailed to include explicit and actionable management strategies.

Following this revision, the updated template was disseminated throughout the wards to facilitate its integration into clinical practice. Finally, comprehensive training and awareness sessions were conducted to familiarize healthcare providers with the revised B-SOAP format, emphasizing its importance in enhancing clinical documentation and continuity of care.

Post-intervention phase

This quality improvement project was conducted through three sequential cycles, each associated with two interventions:

Cycle 1: Sample size: 21 B-SOAP sheets; Intervention: redesigned B-SOAP template; Structured fields for all sections (Background, Subjective, Objective, Assessment, Plan); mandatory data entry prompts; Purpose: improve documentation completeness and standardization.

Cycle 2: Sample size: 25 B-SOAP sheets; Intervention: structured training sessions (2-hour workshops on documentation best practices), audit feedback system (weekly compliance reviews with individualized reports); Purpose: sustain improvements and reinforce adherence.

Cycle 3 (continuous monitoring): Sample size: 22 B-SOAP sheets

Data analysis

The analysis of data was facilitated by Microsoft Excel 2016 (Microsoft Corporation, Redmond, WA), utilizing descriptive statistics such as frequencies and percentages to summarize compliance rates. A comparative analysis was also undertaken to discern patterns and prevalent shortcomings in documentation practices, thereby highlighting opportunities for enhancement.

Ethical considerations

Ethical approval for the study was obtained from the institutional ethics committee of Ribat University Teaching Hospital (approval number: IRB 2021-11-003). Also, to ensure patient confidentiality, all patient-identifiable data were anonymized.

## Results

During the period of the three evaluation cycles, a thorough analysis was performed on a total of 68 B-SOAP follow-up sheets, namely, 21 sheets in the first cycle, 25 sheets in the second cycle, and 22 sheets in the final cycle. As shown in Table 1, significant improvements were noted in overall compliance, which rose from 21.9% during the first cycle to 72% in the second cycle, eventually reaching 90% in the post-intervention cycle. This improvement of 68.1% is consistent with previous studies that highlight the effectiveness and relevance of audit and feedback systems in promoting professional practice and recording patient health [10]. Similar interventions have shown that systematic documentation programs, when combined with periodic evaluations, greatly improve compliance with the quality of medical record keeping [3, 11].

**Table 1:** Compliance with B-SOAP documentation standards across audit cycles B-SOAP: Background, Subjective, Objective, Assessment, and Plan

B-SOAP component	First cycle (21 sheets)	Second cycle (25 sheets)	Third cycle (22 sheets)	Improvement (%)
Background	45.9% (10/21)	65% (16/25)	65% (14/22)	+19.1%
Subjective	18.8% (4/21)	30% (7/25)	40% (9/22)	+21.2%
Objective	5.6% (1/21)	10% (2/25)	20% (4/22)	+16.4%
Assessment	9.0% (2/21)	20% (5/25)	30% (7/22)	+21.0%
Plan	32.5% (7/21)	50% (12/25)	65% (14/22)	+32.5%
Overall	21.9% (21/96)	72.0% (25/35)	90.0% (22/24)	+68.1%

Documentation in the background section demonstrated a significant improvement of 19% (from 45.9% in the first cycle to 65% in the third cycle), while the subjective aspect of the documentation registered an increase of 21.2% (from 18.8% to 40%). On the other hand, the objective section documented an improvement of 16.4%, though with the lowest initial compliance level (from 3.6% to 20%). Similarly, the assessment section registered an improvement of 21.0% (from 9.0% to 30%). On the contrary, the highest improvement across all sections was realized in the planning section with a 32.5% improvement (from 32.5% to 65%). These findings conflict with the current literature that strongly advocates for the use of structured documentation templates and education as the key to ensuring the completeness and accuracy of patient records. These improvements emphasize the need to have iterative audit cycles coupled with structured intervention to maintain the quality of documentation. The improvements noted in the Plan section indicate a stronger emphasis on the development of explicit and actionable management plans. The improvements found in the Subjective and Assessment sections indicate a better identification of the need for intensive clinical assessments. These findings add to the existing evidence that identifies the critical role of continuous monitoring and feedback in the process of quality improvement of clinical documentation. In the future, the continued reinforcement of best practices by workshops and regular audits will be vital to maintaining high levels of compliance and ensuring continued commitment to B-SOAP documentation.

## Discussion

This study explored adherence to the use of a B-SOAP follow-up sheet in the pediatric surgery department of Ribat University Teaching Hospital. Baseline adherence to documentation standards was extremely low, standing at 21.9% (21 out of 96), indicating a chronic inconsistency in recording behavior in the institution. The introduction of a paper-based standardized proforma for a B-SOAP, coupled with focused training sessions and multiple rounds of audits, brought a considerable improvement in adherence to a high of 90% (22 out of 24). The results concur with existing evidence that high-quality audits, when combined with a feedback system, raise the level of clinical documentation standards [1,2].

The results of our study align with the findings of Dolan and Broadbent [12], who demonstrated that the use of structured documentation tools, such as a SOAP-based proforma, significantly improves the quality of clinical records in acute surgical settings. Their study highlighted the importance of standardized formats in reducing documentation errors and enhancing the clarity of patient management plans, which is consistent with our observations in the pediatric surgery context.

Similarly, Mathioudakis et al. [13] emphasized the critical role of maintaining good clinical records for patient safety and continuity of care. Their work supports our findings that structured documentation frameworks, when combined with regular training and audits, can lead to substantial improvements in record-keeping practices, even in resource-limited settings. This is particularly relevant in our study, where the lack of standardized templates and training initially hindered compliance with documentation standards.

Furthermore, Thomas [14] discussed the medico-legal implications of poor documentation, noting that incomplete or inaccurate records can lead to significant issues in patient care and legal accountability. Our study underscores the importance of addressing these gaps through systematic interventions, as evidenced by the marked improvement in compliance rates following the introduction of a revised B-SOAP template and targeted training sessions.

The results of our study concur with Joshi et al.'s work [3], which established that educational interventions, when combined with regular documentation processes, produce remarkable improvement in record quality. Institutions that use electronic health record (EHR) systems would be more consistent and efficient in documentation processes. In a country such as Sudan, where financial, infrastructural, and human resource challenges hinder the adoption of an EHR system, this work highlights the efficacy and practicability of improving documentation processes in a paper-based system. Elhadi Bakheet et al. [5] also found that poor resource availability requires continued reliance on hand procedures, hence justifying the method used in our intervention.

The Plan aspect saw the greatest improvement (+32.5%), indicating that the intervention was useful in reinforcing the focus of constructing particular management strategies. The improvement observed in the Subjective and Assessment areas indicates a more integrated use of patients' data in conjunction with better professional judgment. The incremental improvement observed in the Objective section (+16.4%) shows that making observations standardized is a serious challenge that requires more digital tools in a system that is otherwise resource-constrained.

Our results line up with the body of research on the efficacy of organized documentation templates and instructional interventions when compared to similar studies. Joshi et al. [3], for example, showed that our results are in line with their observations: the use of the SOAP format in pediatric ward rounds considerably improved documentation quality. Emphasizing the importance of training and organized documentation, which supports our method of employing a paper-based system in a resource-limited environment, Koh and Ahmed [4] also underlined the difficulties and advantages of switching to electronic health records in pediatric care.

Consistent with our study's approach and outcomes, Elhadi Bakheet et al. [5] and Muhammad et al. [6] also underlined the need for audits and feedback systems in enhancing surgical documentation. Their efforts at Sudanese hospitals also underlined the difficulties of limited resources, therefore supporting our strategy of running training courses and iterative audits to raise compliance.

Cameron and Rangel [7] spoke on the wider consequences of quality improvement in pediatric surgery and underlined that frequent audits and organized documentation are essential for improving patient safety and quality of treatment. This aligns with our results, where audits, training, and a consistent template combined to greatly increase compliance.

After doing a Cochrane analysis on the impact of audit and feedback on professional practice and healthcare outcomes, Ivers et al. [8] found that these kinds of interventions help to improve documentation procedures quite effectively. The findings of our research confirm this conclusion: organized interventions may significantly increase clinical documentation even in environments with limited resources.

This study has several limitations. First, the study was conducted in a single institution, which may limit the generalizability of the findings. Second, the reliance on paper-based documentation may not be applicable to settings with EHRs. Third, the study duration was relatively short, and long-term sustainability of the improvements was not assessed. Future studies should consider multi-center trials and longer follow-up periods to validate these findings.

## Conclusions

The study at Ribat University Teaching Hospital found that a combination of educational interventions and regular documentation processes can significantly improve clinical documentation quality in resource-constrained environments. The study found that a paper-based standard form, targeted training, and repeated audits led to a 90% increase in compliance, highlighting the importance of clear management strategies. However, the study also acknowledged the need for sustained reinforcement to prevent regression to previous practices and the absence of an EHR system. To sustain these gains, the study recommends updating the B-SOAP template and conducting periodic manual audits.

## Figures and Tables

**Figure 1:**
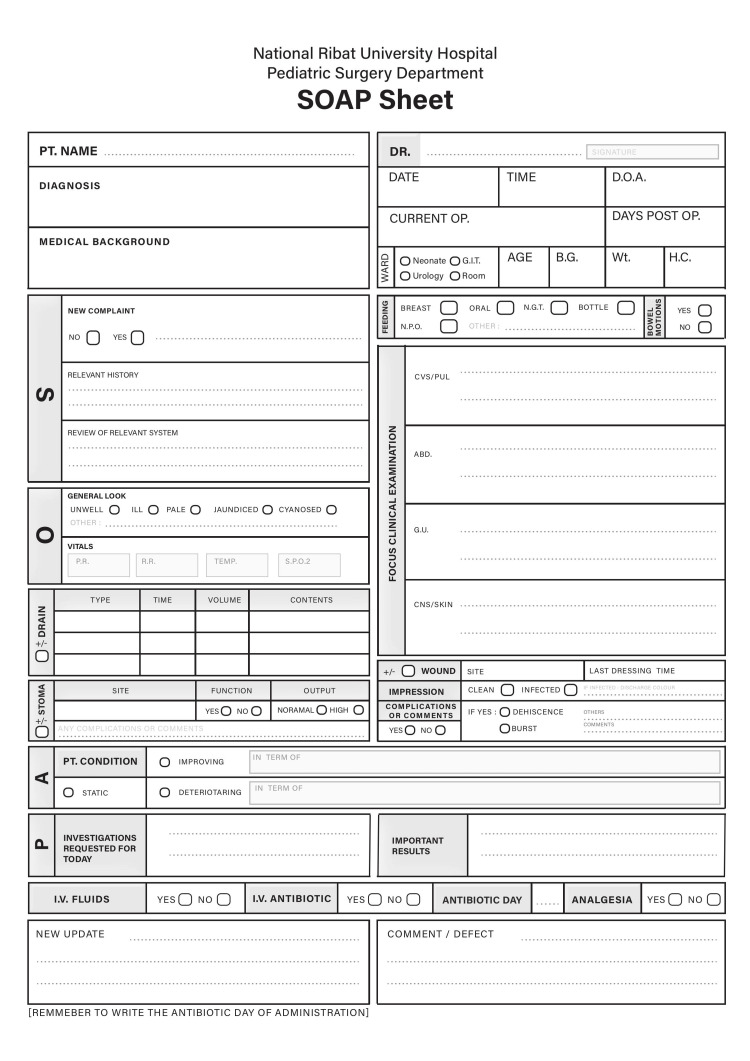
Post-intervention B-SOAP sheet of the pediatric surgery department B-SOAP: Background, Subjective, Objective, Assessment, Plan

## Appendices

Appendix A 

Post-intervention B-SOAP Sheet of the Pediatric Surgery Department
